# Dermal fibroblasts derived from fetal and postnatal humans exhibit distinct responses to insulin like growth factors

**DOI:** 10.1186/1471-213X-7-124

**Published:** 2007-11-07

**Authors:** Kerstin J Rolfe, Alison D Cambrey, Janette Richardson, Laurie M Irvine, Adriaan O Grobbelaar, Claire Linge

**Affiliations:** 1 RAFT Institute of Plastic Surgery, Leopold Muller Building, Mount Vernon Hospital, Northwood Middlesex. UK; 2 Department of Obstetrics and Gynaecology, Watford General Hospital, Vicarage Road, Watford. UK; 3 Department of Plastic and Reconstructive Surgery, The Royal Free Hospital, Pond Street, Hampstead, London. UK

## Abstract

**Background:**

It has been well established that human fetuses will heal cutaneous wounds with perfect regeneration. Insulin-like growth factors are pro-fibrotic fibroblast mitogens that have important roles in both adult wound healing and during development, although their relative contribution towards fetal wound healing is currently unknown. We have compared responses to IGF-I and -II in human dermal fibroblast strains derived from early gestational age fetal (<14 weeks) and developmentally mature postnatal skin to identify any differences that might relate to their respective wound healing responses of regeneration or fibrosis.

**Results:**

We have established that the mitogenic response of fetal cells to both IGF-I and -II is much lower than that seen in postnatal dermal fibroblasts. Further, unlike postnatal cells, fetal cells fail to synthesise collagen in response to IGF-I, whereas they do increase synthesis in response to IGF-II. This apparent developmentally regulated difference in response to these related growth factors is also reflected in changes in the tyrosine phosphorylation pattern of a number of proteins. Postnatal cells exhibit a significant increase in phosphorylation of ERK 1 (p44) in response to IGF-I and conversely the p46 isoform of Shc on IGF-II stimulation. Fetal cells however only show a significant increase in an unidentified 100 kDa tyrosine-phosphorylated protein on stimulation with IGF-II.

**Conclusion:**

Dermal fibroblasts exhibit different responses to the two forms of IGF depending on their developmental maturity. This may relate to the developmental transition in cutaneous wound healing from regeneration to fibrosis.

## Background

It has been known for several decades that human fetuses will heal cutaneous wounds without scarring [1]. Rowlatt's work documented that early human fetuses heal their cutaneous wounds by mesenchymal proliferation resulting in perfect regeneration [1]. Furthermore, although the extracellular matrix (ECM) deposited during fetal wound regeneration is the same composition as that identified in postnatal and adult wound healing they exhibit a distinct difference in the temporal and spatial distribution of each component. For example, the deposition of collagen in fetal repair is indistinguishable from that of the surrounding uninjured tissue whereas in the postnatal human, collagen deposition during wound healing is disorganised [2]. It has therefore been proposed by a number of groups that fetal fibroblasts are the key repair 'effector' cells because it is believed that they dictate collagen deposition, the main component of the dermal ECM [3].

IGF-I is believed to play a role in adult wound healing through its action on fibroblasts and others have shown that the expression of IGF-I and -II increases substantially between days 1–21 post wounding [4,5]. IGF-I plays an important role in cell growth both *in vitro *and *in vivo *[4]; and is known to stimulate fibroblast mitogenesis and ECM synthesis[6]. IGF-I's biological actions are mediated by its receptor, a tyrosine kinase, IGF-IR [7]. Binding of IGF-I to its receptor (IGF-IR) results in receptor autophosphorylation followed by phosphorylation of a number of adaptor signalling proteins such as Shc [7]. Studies have shown that the phosphorylation of Shc followed by MAPKs causes mitogenesis and that IGF-I increases collagen type I mRNA also via the MAPK pathway [8,9].

Treatment of wounds with IGF-I has been shown to accelerate wound healing by the stimulation of fibroblast collagen synthesis, in addition to its mitogenic effect on both keratinocytes and fibroblasts [10,4]. However IGF-I has also been implicated in a range of fibrotic conditions e.g. keloids, hypertrophic scars, Crohn's disease, fibrotic lung disease and glomerular disease [11-14].

Although both IGF-I and IGF-II are expressed in the embryo, IGF-II is thought to have a predominant role early on in development [15]. IGF-II is a member of a small family of genes that have been shown to be subject to genomic imprinting [16,17]. An imprinted gene is expressed primarily from one specific parental allele and such genes have been shown to exert important effects, primarily on fetal development. IGF-II acts through the same receptor as IGF-I, the tyrosine kinase IGF type I receptor, but also binds both the insulin receptor and the IGF type II receptor which is thought to be responsible for IGF-II degradation [18]. DeChiara et al, showed that IGF-II is required for normal fetal growth, as IGF-II null mice were 60% smaller than their wild type littermates [19,20]. However, these growth deficient animals were otherwise apparently healthy and fertile, so IGF-II is not essential for development or survival. Conversely experiments on IGF-I homozygote knockout mice (Igf I^-/-^) have shown that animals are not only reduced in size but display severe muscle dystrophy and most (>95%) died at birth [21]. Clearly therefore both IGF proteins play distinct roles during fetal development and growth. Nevertheless the relative contribution of each IGF during fetal wound healing is currently unknown.

The aim of this study was to determine if early gestational age human fetal dermal fibroblasts (FDF) respond in the same manner as their developmentally mature counterparts (MDF) to the addition of exogenous IGF-I and IGF-II with regard to proliferation, collagen production and finally intracellular signalling. Any differences detected may contribute towards the ability of fetal but not developmentally mature fibroblasts to regenerate the dermis rather than form scar tissue. Our data demonstrates clear differences in cellular responses between the two cell types, with FDF demonstrating minimal mitogenic response to either form of IGF and failing to synthesise collagen in response to IGF-I but not IGF-II. Distinct patterns in the phosphorylation of intracellular proteins in response to IGF stimulus were also identified for FDF and MDF. The contrasting responses of dermal fibroblasts from different developmental stages to these important cytokines may well relate to their dissimilar wound healing responses of either perfect regeneration of dermal architecture or crude filling of the tissue deficit by scar tissue formation.

## Results

### Fibroblast proliferation

**Figure 1:**
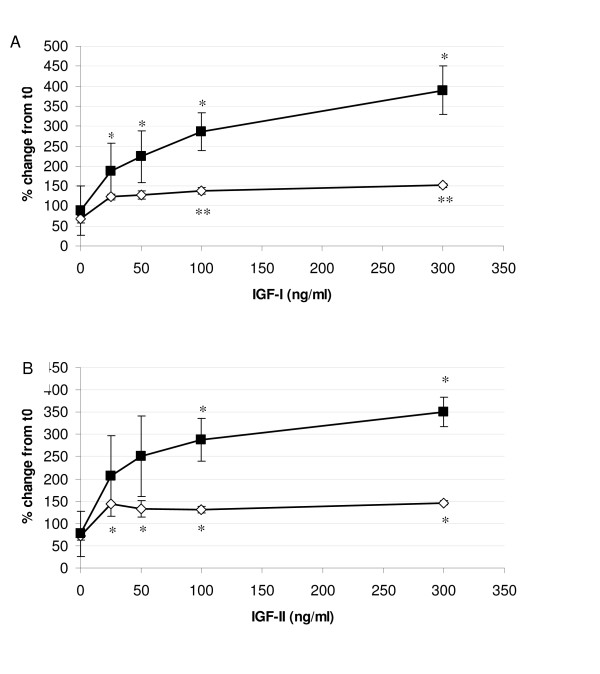
**Proliferation response of dermal fibroblasts to IGF-I and IGF-II**. A: The mean proliferation curve in response to IGF-I at 72 hrs for both FDF (open squares; n = 3) and MDF (closed diamonds; n = 3) in 0.4% FCS-containing medium. Error bars = SEM. (*p = < 0.05; **p = < 0.005). FDF only showed statistical difference at 100 and 300 ng/ml. MDF showed a statistical increase in proliferation at 25 ng/ml, 50 ng/ml, 100 ng/ml and 300 ng/ml compared to untreated controls. B: The mean proliferation curve in response to IGF-II at 72 h for both FDF (open squares; n = 3) and MDF (closed diamonds; n = 3) in 0.4% FCS-containing medium. Error bars = SEM. (*p = < 0.05). FDF showed a statistical increase throughout the concentration range compared to untreated controls. MDF showed a statistical significance from the untreated control at 100 and 300 ng/ml.

A comparison of the proliferative responses to IGF-I and IGF-II by FDF and MDF are shown in Figure 1A and 1B respectively. Identical results were obtained using the WST-1 assay (Figure 1) and the crystal violet proliferation assay (data not shown). MDF demonstrated a clear dose dependent response to IGF-I, with significant increases in cell number above that observed by the untreated control at all concentrations tested (p < 0.05; Figure 1A). The increase in MDF cell number reached a maximum of over four times that of the untreated control at the highest concentration tested (300 ng/ml). In contrast FDF showed a less profound response with a much lower maximal response of twice that of the untreated control, which was reached at a lower concentration of IGF-I at 25 ng/ml, although only became statistically significant at 100 and 300 ng/ml IGF-I (p = 0.0005 and 0.0013 respectively). This response was significantly lower than that exhibited by the MDF cell strains at the two highest IGF-I concentrations tested (100 ng/ml, p = 0.0366 and 300 ng/ml, p = 0.0169).

The proliferative response to IGF-II was almost identical to that of IGF-I, showing an increase in proliferation induced for both cell types although again to a lesser extent by FDF compared to MDF cell strains (Figure 1B). FDF showed a significant increase (p < 0.05) throughout the concentration range used compared to untreated cells, whereas although MDF exhibited a greater response throughout the concentration range this was only significantly different from their untreated controls at 100 ng/ml (p = 0.0500) and 300 ng/ml (p = 0.0350) due to high variability at lower concentrations. Nevertheless the maximal response seen with MDF cell strains was again over four times greater than untreated controls, whereas that seen for FDF strains was significantly lower at 100 (p = 0.0320) and 300 ng/ml (p = 0.0039) being only twice that seen in the untreated FDF control.

### Collagen synthesis

**Figure 2:**
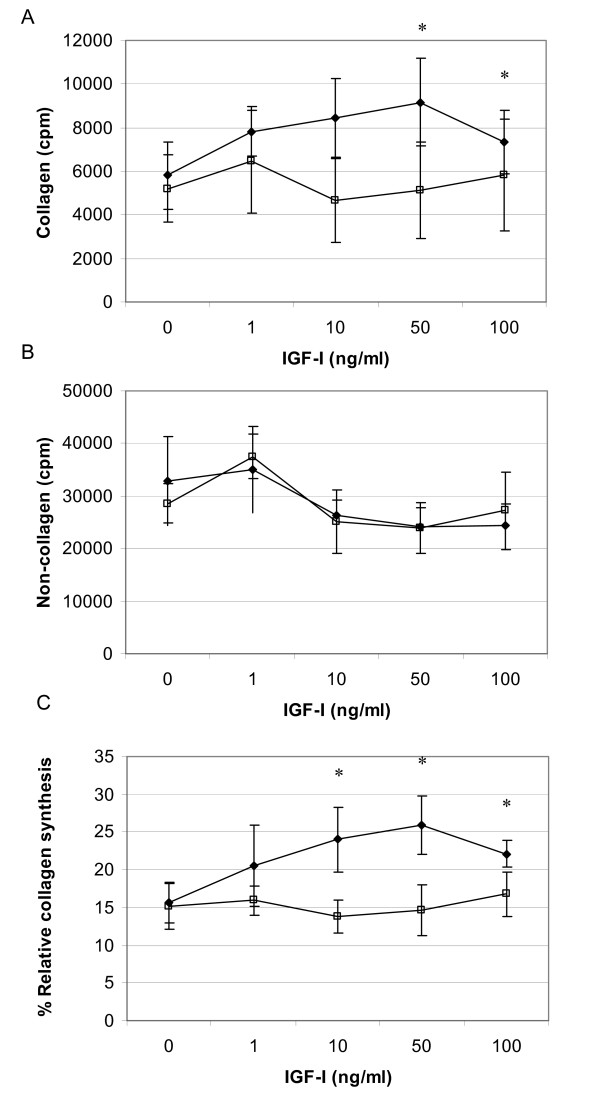
**Collagen production in response to IGF-I**. Radio-labelled proline uptake assay demonstrating modulation of protein synthesis by IGF-I. Panel A and B depict IGF-I elicited changes in collagen and non-collagen synthesis respectively, by FDF (n = 3, open squares) and MDF (n = 3, closed diamonds). All data is represented as cpm/10^5 ^cells (mean ± sem). Panel C illustrates changes in relative collagen synthesis between MDF and FDF in response to IGF-I; data represented as % (mean ± sem). * p = < 0.05. MDF cell strains demonstrated significantly higher collagen synthesis than untreated controls at 50 and 100 ng/ml of IGF-I (*p = 0.034 and 0.047 respectively); and significantly higher %RCS than untreated controls at 10, 50 and 100 ng/ml of IGF-I (*p = 0.047, 0.021 and 0.028 respectively). In contrast FDF cell strains showed no significant effect of IGF-I treatment on either collagen or protein synthesis over the entire titration range.

Modulation of collagen synthesis by IGF-I and II were estimated *in vitro *and the responses between FDF and MDF cell strains compared. IGF-I elicited a dose dependent increase in collagen synthesis by MDF (Figure 2A). Collagen synthesis became maximal at concentrations of 50 ng/ml and over, a 39% (p < 0.05) increase above that elicited by untreated control cells. By contrast, non-collagen protein synthesis did not significantly alter in response to IGF-I, regardless of concentration assessed (Figure 2B). A comparison of the effect of IGF-I on the levels of collagen synthesised as a proportion of total protein produced over the exposure period is shown in Figure 2C. This demonstrates that IGF-1 (10 – 100 ng/ml) significantly and selectively increases collagen synthesis; above any effects on total protein production compared with untreated control cells (p < 0.05). In contrast, FDF did not exhibit any significant response to IGF-I, with regard to either collagen or non-collagen synthesis. Indeed ANOVA analysis of the relative collagen synthesis data determined a statistically significant difference (p = 0.016) in the response of FDF and MDF.

**Figure 3:**
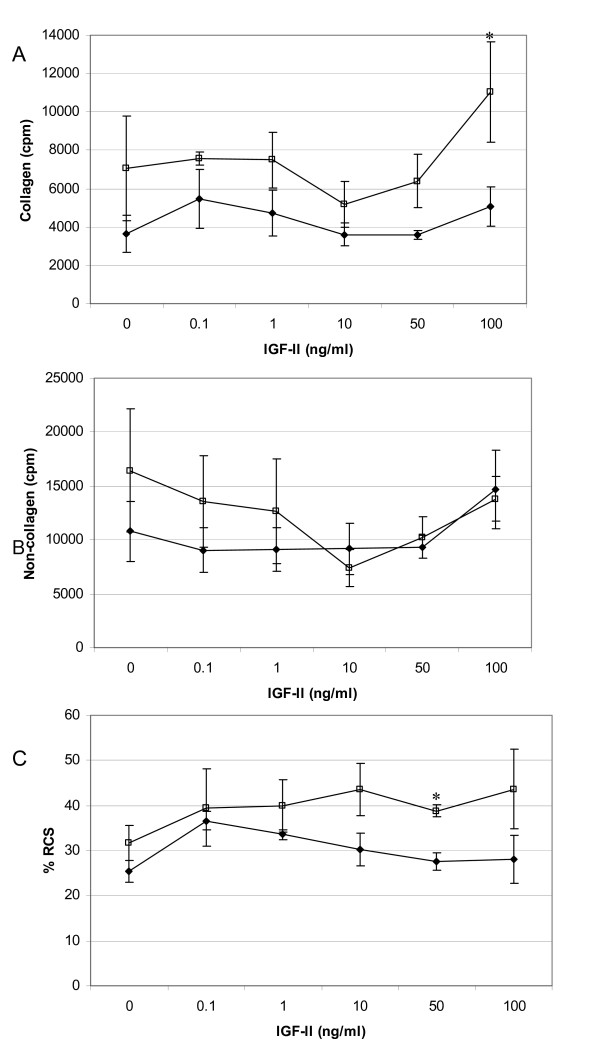
**Collagen production in response to IGF-II**. Radio-labelled proline uptake assay demonstrating modulation of protein synthesis by IGF-II. Panel A and B depict IGF-II elicited changes in collagen and non-collagen synthesis respectively, by FDF (n = 3, open squares) and MDF (n = 3, closed diamonds). All data is represented as cpm/10^5 ^cells (mean ± sem). Panel C illustrates changes in relative collagen synthesis between MDF and FDF in response to IGF-II; data represented as % (mean ± sem). * p = < 0.05. Collagen synthesis was significantly higher for FDF over untreated control at 100 ng/ml IGF-II. There was no significant change in non-collagen synthesis for either cell type. Student t-tests demonstrated that the %RCS produced by FDF with 50 ng/ml IGF-II was significantly higher than that produced by PDF (*p = 0.016). ANOVA analysis showed that both collagen count data and % RCS data from FDF are highly significantly different (p = 0.003 and 0.010 respectively) from PDF cell strain data.

Figure 3 demonstrates the contrasting results obtained with IGF-II, with the only significant increase in collagen synthesis (36% over that of the untreated control) being exhibited by fetal cells at 100 ng/ml (Figure 3A). There was no significant effect on general protein synthesis by either cell type and IGF-II had no significant effect on either total or relative collagen synthesis of MDF over the entire concentration range tested. ANOVA analysis of collagen synthesis as a proportion of total protein synthesis demonstrated that fetal cells show a significantly different relative collagen synthesis over that of MDF (p = 0.010, Figure 3C), with FDF being significantly higher than MDF at 50 ng/ml of IGF-II (p = 0.016).

### Quantification of IGF-I Receptor (IGF-1R)

**Figure 4:**
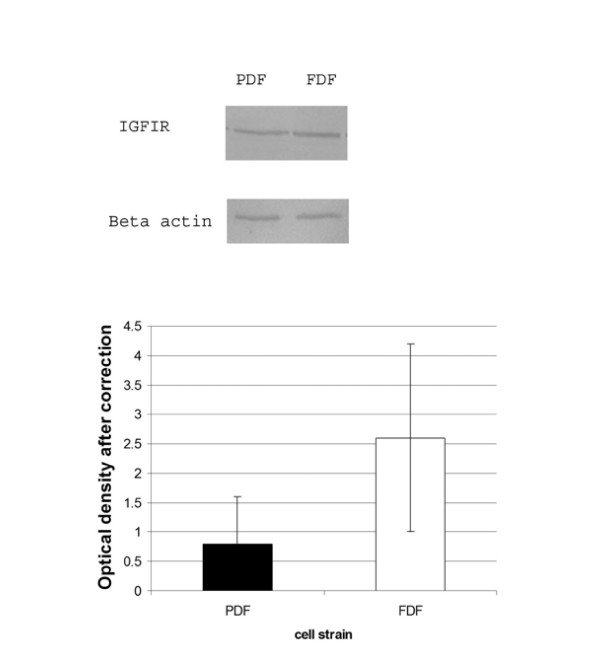
**IGF-IR in dermal fibroblasts**. A: Protein lysates from quiescent cells were analysed by Western blot using a specific antibody against IGF-IR. B: To ensure equal loading β-actin was also used. C: The graph represents the change in IGF-IR between MDF and FDF cell strains (n = 3 for each). Error bars = SEM. No statistical significant difference was demonstrated between the cell types.

Protein lysates from quiescent cells treated with serum free media alone were analysed for expression of IGF-IR using SDS-PAGE and Western blotting using a specific antibody. A typical Western blot for both IGF-IR and loading control together with the graph showing the densitometric analysis of band density after correction for equal loading is shown in Figure 4A–B. Statistical analysis failed to detect any significant difference between the IGF-IR expression of FDF cell strains and MDF (n = 3 each). Fluorescence activated cell sorter (FACS) analysis was also performed (data not shown) on FDF and MDF cell strains (n = 2 of each) to quantify the relative levels of cell surface IGF-IR, and demonstrated no significant difference between the two different cell sources.

### Intracellular signalling

#### Tyrosine phosphorylated proteins

**Figure 5:**
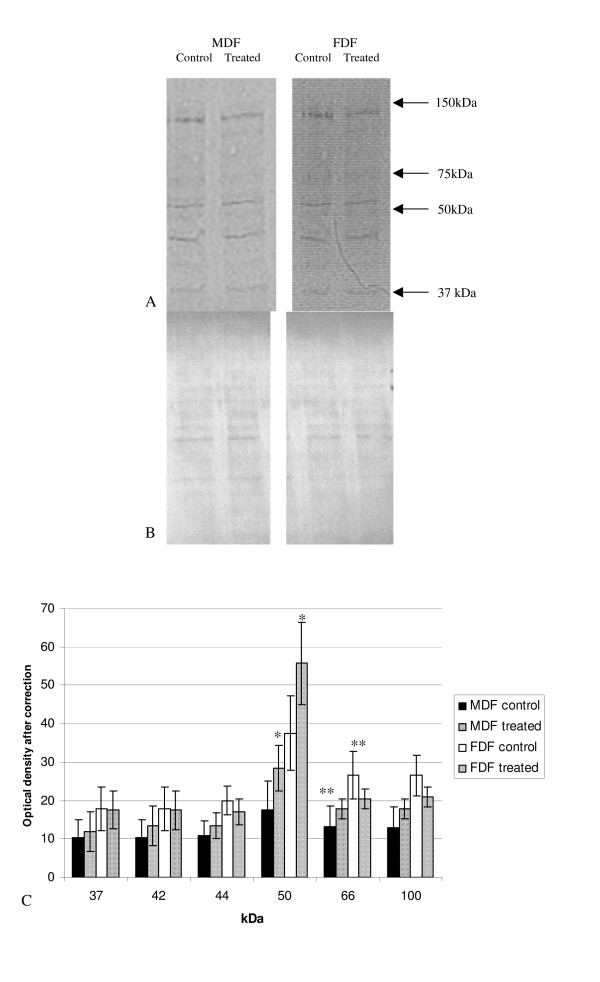
**Tyrosine phosphorylated proteins after stimulation with IGF-I**. A: Protein lysates from cells treated with IGF-I for 20 mins were analysed by Western blot using a specific antibody against tyrosine phosphorylated proteins. B: To ensure equal loading used the MemCode Reversible™ protein stain (Pierce Biotechnology). C: The graph represents the change in tyrosine phosphorylation after ensuring for equal loading. The graph represents the results for FDF (n = 5; closed bar un-stimulated, diamonds represents stimulated cells) and MDF (n = 5 hash bar represents un-stimulated and the closed bar stimulated). Error bars = SEM. A statistical increase was shown in FDF following stimulation with IGF-I compared to MDF at 50 kDa (*p = 0.028); a further statistical difference was shown with an increase of a band at 66 kDa in unstimulated FDF cells compared to unstimulated MDF (**p = 0.024). Two way ANOVA showed a statistical significant (p = 0.007) difference in the mean values among the different cell type greater than would be expected by chance allowing for the difference in treatment.

**Figure 6:**
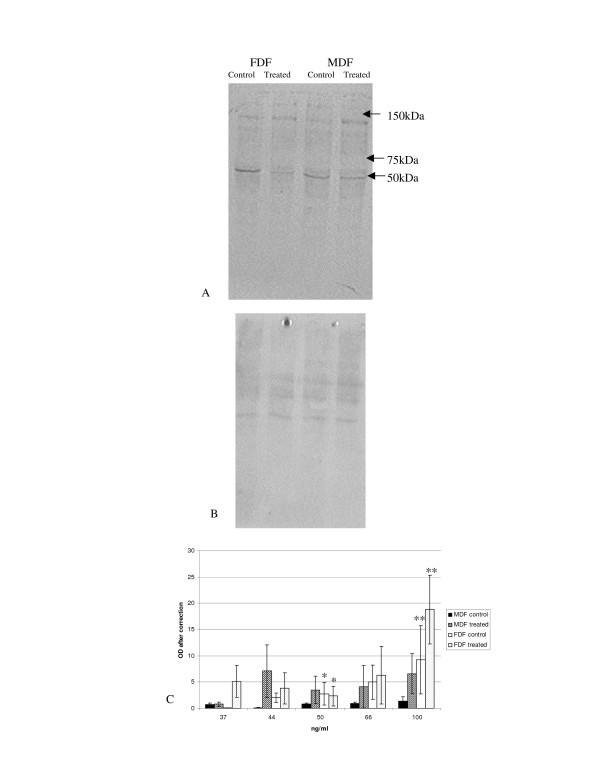
**Tyrosine phosphorylated proteins after stimulation with IGF-II**. A: Representative Western blot. Protein lysates from cells treated with IGF-II for 20 mins were analysed by Western blot using a specific antibody against tyrosine phosphorylated proteins. B: To ensure equal loading used the MemCode Reversible™ protein stain (Pierce Biotechnology) C: The graph represents the mean change in tyrosine phosphorylation after ensuring for equal loading following stimulation with IGF-II for FDF (n = 5 open bar un-stimulated; lined bar represents stimulated lysates) and MDF (n = 5 closed bar represents un-stimulated lysates; thick lines represents stimulated lysates). Error bars = SEM. A statistical decrease in FDF (p = 0.048) was identified for a band at 50 kDa following stimulation with IGF-II. FDF showed a statistical increase when comparing un-stimulated cells with stimulated cells at 100 kDa (p = 0.029) and a further statistical increase was demonstrated between FDF and MDF when cells had been stimulated with IGF-II at for a band at 100 kDa (p = 0.049).

Protein lysates from quiescent cells treated with serum free media alone (unstimulated control) or with the addition of either exogenous IGF-I or IGF-II were analysed by Western blot using an antibody specific for tyrosine phosphorylated proteins. Western blots were assessed by densitometry after ensuring that each sample had equal loading by the use of the MemCode Reversible™ protein stain (Figure 5B). A number of tyrosine phosphorylated protein bands were clearly identified in both untreated FDF and untreated MDF lysates and IGF-I or IGF-II stimulated groups (Figure 5A and 6A respectively). The pattern (number and position) of tyrosine phosphorylated bands was the same for both FDF and MDF cells strains and did not change on stimulation. Although the density of all bands appeared higher in unstimulated FDF compared to MDF, this only reached significance for the 66 kDa band (Figure 5C; p = 0.024).

The density of all the tyrosine phosphorylated protein bands appeared slightly higher in MDF cell strains after stimulation with IGF-I, but were mainly unchanged or lower for FDF cell strains, however none of these differences reached statistical significance (Figure 5C). The only exception to these apparent trends was a band at 50 kDa in FDF cell strains, which increased on IGF-I stimulation, but again this increase failed to reach statistical significance. Despite the similar tendency of this band to increase in density on IGF-I stimulation in MDF and FDF strains alike, that of MDF cells was significantly lower than that of fetal cells (p = 0.028).

Both FDF and MDF demonstrated a trend toward increased density of most tyrosine phosphorylated protein bands after IGF-II stimulation (Figure 6). This increase in density on stimulation was statistically significant (p = 0.029) for the 100 kDa band seen in FDF lysates, but failed to reach statistical significance for all bands detected in the MDF strain lysates, The increased tyrosine phosphorylation of this 100 kDa band in FDF strains was also significantly higher than that induced in MDF (p = 0.049). An exception to this overall increase in band density on stimulation was a tyrosine-phosphorylated band at 50 kDa in FDF cell strains, which was significantly reduced (p = 0.048).

#### Shc

**Figure 7:**
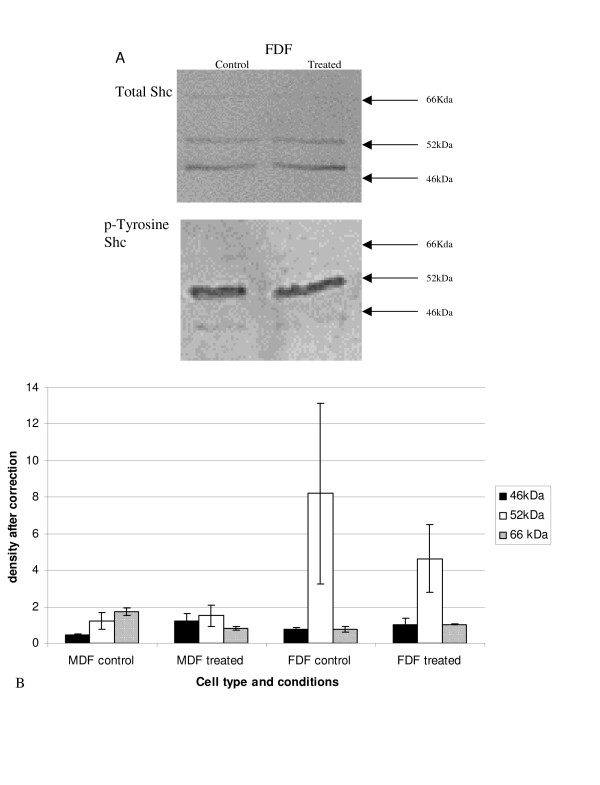
**Tyrosine phosphorylated Shc after stimulation with IGF-I**. A: Protein lysates from cells treated with IGF-I for 20 mins were analysed by Western blot. A: Shows a representative western blot analysis for total Shc. Protein lysates were immunoprecipiated with an anti-Shc antibody as described in the materials and methods and the precipitate was run on a western blot and stained with the antibody p-Tyr and a representative western is shown in B. B: A graph representing the densitometry, after correcting for total-Shc. Bars show average for n = 5 FDF and n = 5 MDF and SEM (closed bar 46 kDa, open bar 52 kDa, and hashed bar 66 kDa). No statistical difference was demonstrated for any group.

Protein lysates were immunoprecipitated, with an anti-Shc antibody and the precipitate electrophoresed and analysed by Western blotting with an anti-tyrosine phosphorylated antibody. Three bands were identified, which corresponded to the three isoforms of Shc, 46 kDa, 52 kDa and 66 kDa (Figure 7A).

The apparently higher levels of tyrosine phosphorylated 52 kDa Shc exhibited by unstimulated FDF strains compared to both IGF-I stimulated FDF cells and MDF cells irrespective of stimulation did not reach statistical significance. The other isoforms clearly showed no difference between cell strains or between IGF-I treated and untreated control. On combining data for the similar acting Shc isoforms (p46 and p52), the elevated expression of p46/p52 seen for untreated FDF, over both untreated MDF and IGF-I treated FDF still failed to reach statistical significance.

**Figure 8:**
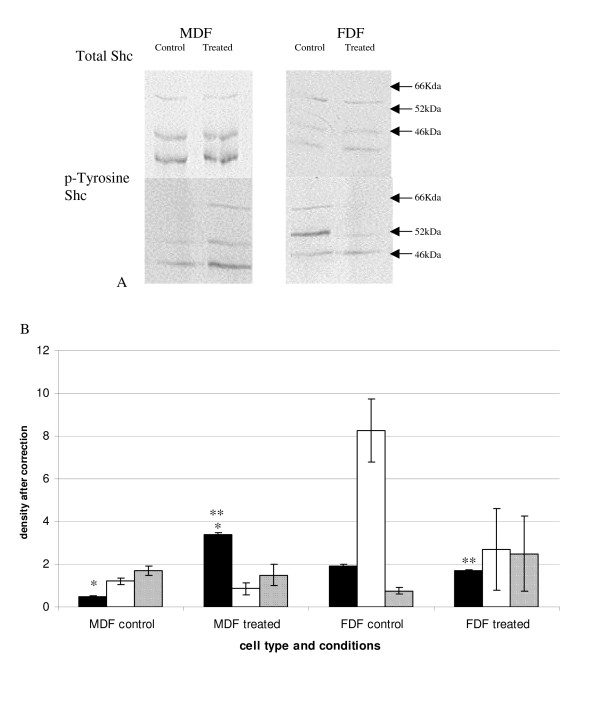
**Tyrosine phosphorylated Shc after stimulation with IGF-II**. Protein lysates from cells treated with IGF-II for 20 mins were analysed by Western blot. A: Shows a representative western blot analysis for total Shc. Protein lysates were immunoprecipiated with an anti-Shc antibody as described in the materials and methods and the precipitate was run on a Western blot and stained with the antibody p-Tyr and a representative Western blot is shown in A. B: shows the graph plotting the densometry, after correcting for total Shc. A statistical significant difference was demonstrated for p46 with MDF showing a significant increase after treatment (*p = 0.03) and the MDF treated group was statistically greater than the FDF treated group (**p = 0.004). n = 5 FDF and n = 5 MDF (closed bar 46 kDa, open bar 52 kDa, and hashed bar 66 kDa). Error bars = SEM.

Protein lysates from cells stimulated with exogenous IGF-II were also immunoprecipitated with an anti-Shc antibody and analysed by Western blotting as described previously. FDF and MDF showed different patterns of Shc phosphorylation (Figure 8), with MDF showing a significant increase in the phosphorylated form of p46 with stimulation (p = 0.03) whereas FDF did not. Indeed there was significantly lower phosphorylated p46 in stimulated FDF compared to stimulated MDF (p = 0.004). Unstimulated FDF cells in this experiment also showed the same elevated p52 as that demonstrated in the IGF-I experiment, though again this did not reach statistical significance between either cell strain types or treatments. Combining data for the similar acting p46 and p52 isoforms, there was again no statistical significance between cell strains or between untreated and IGF-II treated samples.

#### p-ERK

**Figure 9:**
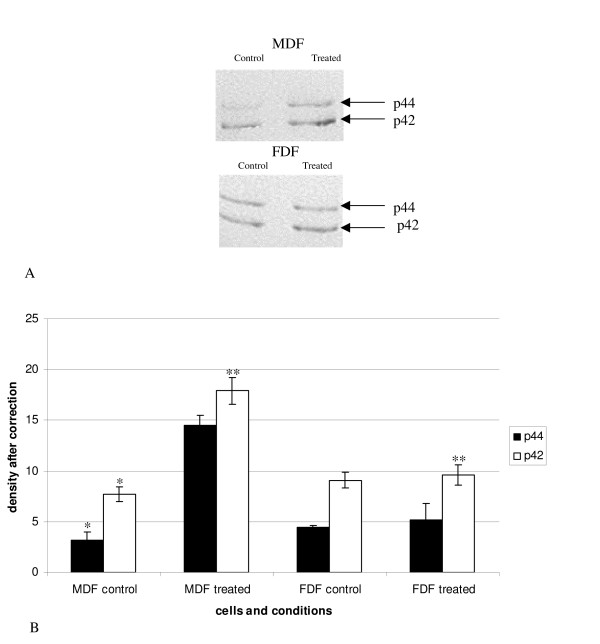
**Phosphorylated MAPK in response to IGF-I**. Protein lysates from cells treated with exogenous IGF-I were analysed by Western blot analysis using a specific anti MAPK (Promega). A: shows a representative Western blot. B: shows a graph representing n = FDF, n = MDF with error bars SEM. A statistical significant increase was demonstrated for p44 in the MDF control (untreated group) and the MDF treated group (*p = 0.04). A trend towards a significant increase was also demonstrated between MDF control and MDF treated for p42 (p = 0.07). FDF showed no significant increase. A statistical significant difference was identified between stimulated FDF and stimulated MDF for p42.

Protein lysates were electrophoresed and analysed by Western blotting which was probed with an anti-p-ERK antibody. MDF significantly increased phosphorylated p44 on IGF-I stimulation (p = 0.04; Figure 9). A similar trend was demonstrated for phosphorylated p42, however this just failed to reach significance (p = 0.07). In contrast, the pattern of phosphorylation of both ERK isoforms in FDF did not alter on stimulation with IGF-I. Indeed, the amount of phosphorylated p42 detected in IGF-I-treated FDF cells was shown to be significantly lower than that seen in stimulated MDF cells (p = 0.04).

Stimulation with IGF-II showed no change in ERK phosphorylation in MDF following stimulation whereas FDF showed a fall in the phosphorylation of ERK with stimulation though this did not reach statistical significance (data not shown). The level of phosphorylated p44 in MDF stimulated with IGF-II was higher compared to stimulated FDF though this failed to reach statistical significance (p = 0.061).

## Discussion

The biological actions of the IGFs are well defined and include substantial effects on growth, differentiation or apoptosis in a number of cell types, including fibroblasts, and myofibroblasts [22-28]. Both forms of IGF are substantially up regulated during adult wound healing and IGF-I at least is thought to play a prominent role via direct effects on dermal fibroblasts [4,5,10]. Given the presence of these cytokines throughout cutaneous wound healing and their implication in fibrosis of other tissues, we have compared their effects on dermal fibroblasts derived from two developmentally distinct sources that differ in their predisposition towards cutaneous scarring [11-14]: early gestational age human fetuses (where scar-free wound healing is achieved) and developmentally mature tissue (where dermal wound healing inevitably results in scar formation). Our study has shown that dermal fibroblasts derived from these different sources (fetal – FDF and mature – MDF) differ in the extent of their proliferative response to both forms of IGF (-I and -II). Both IGF-I and -II significantly induce the proliferation of MDF (after 72 hrs up to four times that of untreated cells), whereas FDF showed a significantly lower proliferative response reaching a maximum of only double that of the untreated control. These results suggest that human dermal fibroblasts derived from early gestational fetuses exhibit a lower response to the mitogenic (and therefore potentially profibrotic) properties of these important wound healing cytokines. As far as the authors are aware this is the first publication that shows the effects of these IGFs on dermal fibroblasts derived from a non-scarring phenotype (early gestational age human fetus) and a scarring phenotype (mature). Other studies published comparing the effects of IGFs on "fetal" versus adult fibroblasts appear to contradict our findings, however these studies used more developmentally mature tissue sources (late gestational age fetus or newborn respectively) rather than the non-scarring early gestational age fetal cells used here [29,30]. Interestingly a similar reduction in proliferative response has also been reported after stimulation with TGF-β1, by early gestational age (14d) murine dermal fibroblasts compared to later gestational age samples [31].

IGF-I has also been shown to stimulate collagen production in a number of developmentally mature tissues and cells including fibroblasts and myofibroblasts [7,32-36]. In agreement, our study has demonstrated that MDF are stimulated to synthesise collagen, both total and relative to total protein synthesis, in response to IGF-I. In contrast however, FDF cells failed to show any increase collagen synthesis in response to IGF-I. A similar tendency of developmentally immature cells and tissue exhibiting reduced collagen synthesis has been suggested in other experimental systems, both *in vivo *and *in vitro *[37,38] ; the latter being reported in response to stimulation with exogenous TGF-β1 [31,39]. Indeed morphometric studies using *in situ *hybridization of rabbit tissue suggest that although collagen expression does increase on wounding in the early fetus, that this is simply through an increase in cell number within the wound site [40]. Adult wounds, however, increase collagen synthesis by both fibroblast migration and an induction of increased collagen expression by individual cells.

Collagen synthesis (both total and relative) resulting from stimulation with IGF-II was less clear-cut, but converse to the IGF-I results appeared significantly induced in FDF cells, with MDF cells exhibiting no significant response over the entire titration range of this cytokine. Although Grotendorst et al also established that IGF-II failed to stimulate collagen synthesis in developmentally mature cells (rat kidney fibroblasts), this absence of response may also be tissue specific since Murphy et al demonstrated that IGF-II but not IGF-I increased collagen synthesis in healing adult rabbit tendons [41,42].

Interestingly our results suggest a potential developmental switch in the response of dermal fibroblasts to these growth factors that is mirrored by known changes in their relative expression during embryonic development, where circulating levels of IGF-I are reported to be low in the fetus and increase throughout gestation, whereas IGF-II predominates in fetuses [43-45].

The IGF-I receptor, IGF-IR, is a tyrosine kinase that mediates IGF-I's biological actions, any changes of which would affect cellular responses [7]. However, Western blotting of total protein levels of IGF-IR and FACS analysis of cell surface levels of IGF-IR demonstrate identical expression by both MDF and FDF.

Cell regulation and cell responses to growth factors such as the IGFs are relayed by a number of pathways involving intracellular signalling proteins, often involving alteration of the phosphorylation state of tyrosine, serine, or threonine residues on a number of intracellular signalling proteins [46-48]. Furthermore, a close correlation exists between increased tyrosine phosphorylation and increased activity of a number of receptor tyrosine kinases such as the IGF-I receptor. Work to date on the role of tyrosine phosphorylation in fetal regeneration has solely used animal models e.g. rat, where five tyrosine phosphorylated protein bands were identified in quiescent fetal dermal fibroblasts (170, 130, 66, 52, 46 kDa) whereas postnatal specimens showed only one band (190 kDa) [38]. However, further work by Chin et al on human dermal fibroblasts from developmentally mature tissue demonstrated the same pattern of five bands as that seen in fetal rat cells [49]. In addition, our work using a commercially available antibody and cells that had been quiescent for 72 hrs demonstrated comparable patterns of multiple phosphorylated protein bands ranging from 37–250 kDa in both FDF and MDF in the absences of stimulation with either of the IGFs. On stimulation with IGF-I we saw no significant change in either the band pattern or band densities in either MDF or FDF cell preparations. Similarly, Chin et al also found no enhancement of the tyrosine phosphorylation pattern for the five bands identified in quiescent dermal fibroblasts on stimulation with serum [49]. On stimulation with IGF-II we did not detect any change in banding pattern or density in MDF, however FDF showed a statistically significant increase in the density of a 100 kDa band along with a slight but significant decrease in the density of a 50 kDa band.

We went on to examine specific signalling proteins known to be activated by IGF's and to be required to elicit either their proliferative or collagen synthesis response [8,9]. The adaptor protein Shc is expressed as three known transcripts with differing effects on function; Shc p52 and p46 are found in every cell type with an invariant reciprocal relationship, whereas p66 expression varies between cell types and is sometimes absent [50]. Shc isoforms are tyrosine phosphorylated after activation by a large number of tyrosine kinase receptors e.g. that of insulin, IGFs, epidermal growth factor, platelet derived growth factor and fibroblast growth factor [51,52]. Activated p46/52 isoforms transmit signals to other signalling proteins (e.g. Ras) and have been implicated in the cytoplasmic propagation of mitogenic signals in a number of cell types including fibroblasts[53]. It remains unclear if p52 and p46 have functional differences, whereas p66 is distinct being part of the complex inhibitory-stimulatory network that converges on growth factor regulated genes, e.g. fos [54]. The relative positive/negative signal balance of the three Shc isoforms within different source cells could determine differential cellular responses to IGFs.

Surprisingly neither MDF nor FDF exhibited any statistically significant differences in the phosphorylation of all three Shc isoforms following stimulation with IGF-I. Perhaps of note is the observed trend of higher phosphorylation of the p52 form of Shc in FDF compared to MDF, which appeared to be reduced on treatment with both IGF-I and -II. Nevertheless, although these differences were reproducible, the high variability demonstrated between individual strains meant that they failed to reach statistical significance. Stimulation of MDF with IGF-II gave a significant increase in the phosphorylated form of the Shc isoform p46, which was also significantly greater than that seen in stimulated FDF, whereas the other two isoforms did not significantly change. FDF showed no significant change in any Shc isoform with stimulation of exogenous IGF-II. The pattern of phosphorylation of Shc isoforms does not correspond with the pattern of the 50 kD band seen in the phosphorylated tyrosine Western blots.

Another signalling event associated with IGF-R1 function is the activation of MAP kinase (ERK1 and ERK2) and subsequent translocation into the nucleus, where it mediates the phosphorylation of specific transcription factors, and progression through the cell cycle [55-59]. In addition, MAP kinase activity has been shown to be required for the IGF-I-induced increased expression of collagen α1 (I) in rat intestinal epithelial cells[9]. Our data has shown that on stimulation with IGF-I quiescent MDF show an increase in the phosphorylation of the ERKs, which was statistically significant for p44 (ERK1). Whereas FDF cell strains showed no change in phosphorylation pattern, which is consistent with the reduced mitogenic response and lack of induction of collagen synthesis seen on stimulation with IGF-I. Neither FDF nor MDF showed any significant increase in phosphorylation of ERKs after induction with exogenous IGF-II. Others, studying different cell types have suggested that IGF-II can activate ERK in keratinocytes, the C2C12 cell line and the extravillous trophoblast [60-62]. Work is on-going to assess the phosphorylation of ERK in response to IGF-II over a longer and more comprehensive time course. The differing outcome between IGF-I and IGF-II in the tyrosine phosphorylation of proteins in FDFs compared to MDF may well be indicative of a developmentally related change in response to these growth factors.

In summary human fetal dermal fibroblasts appear to respond differentially to the two IGFs and in an apparently converse manner to that of their developmentally mature counterparts. FDF exhibit a significantly lower mitogenic response to both IGF-I and -II than that seen with MDF, and furthermore unlike MDF cell strains, clearly fail to synthesise collagen in response to IGF-I. In contrast to MDF cell strains, fetal cells do appear to demonstrate a slight induction (~30% of unstimulated levels) of collagen synthesis in response to IGF-II. FDFs do not appear to increase the tyrosine phosphorylation of a number of proteins including Shc and ERKs in response to IGF-I over the time course used. However, FDF do appear to respond to IGF-II by significantly increasing the density of an as yet unidentified 100 kD tyrosine-phosphorylated protein and reducing the density of a 50 kD tyrosine-phosphorylated protein, though again neither Shc nor ERK showed any increase in activation with treatment. In contrast MDF cell strains exhibited no significant difference with stimulation of either form of IGF in the general amount of tyrosine phosphorylated proteins, although significantly increased phosphorylation of the p46 form of Shc after IGF-II stimulation and in the p44 form of ERK after IGF-I stimulation. Work is ongoing to further study the signalling pathways involved in the response of FDF to IGF-I and -II. It may be possible in future to switch off the specific response of postnatal cells to IGF-I, thus making their behaviour more like that seen with fetal dermal fibroblasts. Manipulation of cell behaviour from postnatal to fetal with regard to these cytokines may result in improvement of scarring.

## Conclusion

Dermal fibroblasts exhibit different responses to the two forms of IGF depending on their developmental maturity. This may relate to the developmental transition in cutaneous wound healing from regeneration to fibrosis.

## Methods

### Reagents

Recombinant growth factors IGF-I, IGF-II from R&D systems (UK). Antibodies p-Tyr (PY99), Shc (PG-797), IGF-IR (Sc 463, Sc 462 Santa Cruz, California, USA), Anti-Active^® ^MAPK (Promega, Southampton, UK), β-Actin (Abcam, Cambridge UK). Nitrocellulose membranes were purchased from Amersham Life Technologies (Bucks, UK). Normal anti-rabbit and anti-mouse conjugated peroxidase immunoglobulins were purchased from Dako (Ely, Cambridgeshire, UK). WST- 1 (Roche, Diagnostics Ltd, Welwyn Garden City, Herts UK). Memcode reversible protein stain (Pierce Biotechnology, Cramlington, Northumberland, UK). All tissue culture products were from Gibco (Paisley, UK) unless specified.

### Cell culture

Human dermal fibroblasts both fetal (obtained from elective termination of pregnancies following local ethical approval; gestation <14 weeks; FDF, n = 7 all digits) and developmentally mature fibroblasts (MDF, n = 7 from infants <2 y, digits; n = 3 from children age 4–10 y from corrective ear surgery and n = 3 from adult from patients undergoing elective reconstructive surgery, Breast reductions) were established by explanting tissue. No differences were seen in behaviour of cell from different body sites. Cells were grown in Dulbecco's modified Eagle's medium (DMEM) supplemented with 10% fetal calf serum (FCS), 2 mM glutamine and 100 U/ml penicillin and 100 mg/ml streptomycin (NGM) unless otherwise specified. All experiments were performed on passage 1–6 cells. Human recombinant IGF-I and IGF-II were used at 100 ng/ml unless specified otherwise.

### Cell proliferation

Fibroblasts were seeded at 5 × 10^3^/well into a 96-microtitre plate (Greiner, Stonehouse, Gloucestershire, UK) and allowed to attach overnight in minimum media (DMEM supplemented with 0.4% FCS) to maintain cells in a healthy but quiescent state. After, 24 hours, the media was aspirated and replaced by test media (minimum media with serial dilutions of IGF-I and -II) and cultured for a further 72 hours. Cell number was assessed using WST-1 assay (Roche, Lewes, UK). Briefly 10 μl WST-1 solution was added to each well. The cells were incubated for 1 hour in standard tissue culture conditions. The absorbance, which related to the number of viable cells converting the reagent to coloured formazan crystals, was read at 450 nm on a Bio-Rad Plate reader. Experiments were performed in triplicate on FDF and MDF derived cell strains (n = 3 for each).

The WST-1 assay results were validated by using an alternative colourimetric assay based on the uptake of crystal violet dye. The cells were fixed and stained with the crystal violet/fix solution (0.5% crystal violet, 5% formol saline, 50% ethanol, 0.85% NaCl) for 10 min at room temperature and washed ×3 with PBS. The dye taken up by the cells was then eluted using 100 μl of 33% acetic acid for 10 mins and the colour read at 540 nm on a Bio-Rad plate reader. Wells not containing cells were used to correct for background staining. Experiments were performed in triplicate on 3 cell lines for FDF and MDF (not shown). Both methods gave identical results; however the WST-1 assay showed considerably less variation.

### Collagen synthesis

Collagen synthesis was assessed via incorporation of radiolabelled proline. This method allows comparison of the effects of IGF's on synthesis of secreted collagen and de novo protein production. Confluent fibroblast monolayers were prepared in 24-well plates, cultured in pre-incubation media: DMEM supplemented with 2 mM L-proline, 50 μg/ml ascorbic acid, 2% FCS, 1 U/ml penicillin/streptomycin, 2 mM L-glutamine and 7 mM Hepes. Twenty-four hours later, the media was replaced with pre-incubation media supplemented with 5 μCi/ml ^3^H L-proline [2,3,4,5] (New England Nuclear, a division of Perkin Elmer, UK) and serial dilutions of IGF-I and II (1–100 ng/ml). After a twenty-four hour exposure period, media was collected for analysis and total cell number per well assessed by trypan blue exclusion using a haemocytometer. All treatments were conducted in replicates of four and the experiment repeated with each of three cell lines (FDF and MDF respectively).

Determination of collagen and non-collagen synthesis in culture media was evaluated in a modification of a well-defined micro assay as previously described by Mariotti et al [63]. Synthesis of collagen and non-collagen protein was expressed respectively as collagenase-soluble and collagenase-insoluble counts per minute (cpm). A correction factor of 5.4 for non-collagen protein was used to adjust for the relative abundance of proline and hydroxyproline in collagen [64]. The resulting counts per minute for collagen and non-collagenous protein production were then normalised against viable cell number in each well and expressed as cpm/10^5 ^cells. Relative collagen synthesis (RCS) was calculated as the amount of collagen synthesised as a proportion of the sum of collagen and non-collagen synthesis, expressed as a percentage.

### Western blotting

Fibroblasts (1 × 10^6 ^cells) were cultured in serum free media (DMEM with 2 mM glutamine and antibiotics 100 U/ml penicillin and 1 mg/ml streptomycin; SFM) for 72 hrs. The cells were treated with either IGF-I or IGF-II for 20 mins or left in SFM (untreated control).

Cultures (1 × 10^6 ^cells) were rinsed twice with ice cold PBS and then lysed in ice-cold buffer (50 mM Tris-HCL pH 6.8, 0.1% SDS, 1 mM phenylmethylsufonyl fluoride, 2 mM sodium orthovanadate, 1 μg/ml aprotinin). Cell lysates were scraped using a rubber policeman from the dishes and repeatedly pipetted to shear DNA. The lysates were then incubated on ice for 10 mins prior to centrifugation at 12,000 rpm for 10 mins to remove insoluble material. Volumes of lysate equivalent to equal protein were electrophoresed on a 10% sodium dodecyl sulphate-polyacrylamide gel and transferred to a nitrocellulose membrane using a Bio-Rad Mini Trans-Blot Cell. Membranes following transfer were stained with MemCode™ reversible protein stain as per manufacturer's instructions to ensure equivalent loading of protein. Membranes were then blocked with Tris buffered saline containing 3% bovine serum albumin and 0.1% polyoxyethylene-sorbitan monolaurate (Tween 20; Sigma, Gillingham, Dorset, UK) for 1 hr. The membranes were washed in Tris buffered saline/0.1% Tween 20 (TTBS) and then incubated with the appropriate primary antibody for 2 hrs at room temperature. Blots were then washed in TTBS and then incubated in the appropriate secondary antibody conjugated to alkaline phosphatase for 1 hr at room temperature. The membranes were then washed and developed in Vectar Blue Substrate (Peterborough, UK) to visualise immunoreactive bands. All western blots were repeated in triplicate for 5 FDF and 5 MDF cell lines.

Band density was quantified by scanning densitometry using the UVP system. Density readings were corrected for variations in loading, with activated (phosphorylated) Shc being compared to total Shc, IGF-IR normalised with β-actin and all other proteins of interest compared to the MemCode Reversible™ (Pierce Biotechnology) protein stain. Data presented graphically is the mean of all 5-cell strains per tissue type.

### Immunoprecipitation

Following treatment with IGF-I or IGF-II cultured quiescent human dermal fibroblasts (1 × 10^6 ^cells) were washed with ice-cold PBS and then lysed in ice-cold lysis buffer as described previously. Cell lysates were pre-cleared by incubation with protein G-Sepharose beads at 4°C for 30 mins. The beads containing the lysates were centrifuged and supernatants collected. Anti-Shc antibody was added and incubated overnight at 4°C. Immunocomplexes were collected by centrifugation and the supernatants discarded. The beads with the immunocomplexes were washed with lysis buffer, mixed with Laemmli sample buffer, boiled for 5 mins and resolved on an SDS-polyacrylamide gel electophoresis. Western blotting was performed using an anti-phosphotyrosine antibody as described earlier.

### Fluorescence activated cell sorter (FACS)

Cells were placed in single cell suspension with the use of accutase and washed with PBS. Cells (10^6^) were incubated with 1 μg/ml of a mouse monoclonal antibody specific for IGF-I receptor (3B7, sc-462 Santa Cruz Biotechnology, Inc) or an isotype control (IgG1, sc-2866 Santa Cruz Biotechnology) for 90 min at 4°C. Cells were then washed in PBS and incubated with fluorescein isothiocyanate-conjugated anti mouse for 1 hour at 4°C. Cells were then washed again in PBS prior to FACS analysis. Data was accumulated from 5000 cells and gated to remove debris/cell aggregates. The intensity of fluorescence was quantified in two ways; both the mean or mode fluorescence corrected for background (isotype control sample) was calculated and compared between n = 2 each of either FDF or MDF cell strains. All four-cell strains were stained and analysed simultaneously to allow comparison.

### Statistical analysis

Statistical significance was analysed using Student t-test and/or ANOVA followed by all pair wise multiple comparison procedures (Tukey test) where appropriate. Values of p < 0.05 were considered significant. Sigma Stat 2.0 software was used for all statistical analysis.

## Abbreviations

ECM: Extra cellular matrix

ERK: Extra cellular signal regulated kinase

FCS: Fetal calf serum

FDF: Fetal dermal fibroblasts

IGF-I: Insulin like growth factor -I

IGFIR: Insulin like growth factor-I receptor

IGF-II: Insulin like growth factor II

MAPK: Mitogen activated protein kinase

NGM: Normal growth media

MDF: Postnatal dermal fibroblasts

SFM: Serum free media

## Authors' contributions

KJR: Establishment of most cell strains and all practical work with the exception of the radioactive collagen assay, analysed data and wrote the paper; JR: Assisted with all practical work except radioactive collagen assay; AC: Performed the radioactive collagen assay and established some cell strains; LI: Provided the fetal tissue; CL and AOG: analysed the data and helped draft the paper. All authors read and approved the final manuscript.
